# Physical education Teachers’ and public health Nurses’ perception of Norwegian high school Students’ participation in physical education – a focus group study

**DOI:** 10.1186/s12889-015-2660-y

**Published:** 2015-12-24

**Authors:** Eirik Abildsnes, Tonje H. Stea, Sveinung Berntsen, Christina S. Omfjord, Gudrun Rohde

**Affiliations:** Department of Global Public Health and Primary Care, University of Bergen, Bergen, Norway; Department of Public Health, Sport and Nutrition, University of Agder, Kristiansand, Norway

**Keywords:** Physical education, Focus group, Lifestyle, Self-determination

## Abstract

**Background:**

High quality physical education programs in high schools may facilitate adoption of sustainable healthy living among adolescents. Public health nurses often meet students who avoid taking part in physical education programs. We aimed to explore physical education teachers’ and public health nurses’ perceptions of high school students’ attitudes towards physical education, and to explore physical education teachers’ thoughts about how to facilitate and promote students’ participation in class.

**Methods:**

Prior to an initiative from physical education teachers, introducing a new physical education model in two high schools in the South of Norway, we conducted focus groups with 6 physical education teachers and 8 public health nurses. After implementation of the new model, we conducted two additional focus group interviews with 10 physical education teachers. In analyses we used Systematic Text Condensation and an editing analysis style.

**Results:**

In general, the students were experienced as engaged and appreciating physical education lessons. Those who seldom attended often strived with other subjects in school as well, had mental health problems, or were characterized as outsiders in several arenas. Some students were reported to be reluctant to expose their bodies in showers after class, and students who seldom attended physical education class frequently visited the school health services. Although the majority of students were engaged in class, several of the students lacked knowledge about physical fitness and motoric skills to be able to master daily activities. The participants related the students’ competence and attitude towards participation in physical education class to previous experiences in junior high school, to the competence of physical education teachers, and to possibility for students to influence the content of physical education programs.

**Conclusions:**

The participants suggested that high school students’ attitudes towards participation in physical education is heterogeneous, depends on the students’ previous experiences, and on their present health and quality of life. All participants recommended adolescents to take part in program development, and selecting activities that generate competence, fun and enjoyment.

## Background

Regular physical activity (PA) is beneficial to physical, mental and social aspects of health among adolescents [1, 2]. Physical inactivity increases the risk of major non-communicable disease, shortens life expectancy, and substantially increases the cost of health care spending [3, 4]. Globally, 80.0 % of adolescents do not reach the recommended levels of PA [5]. In Norway 87.0 % of girls and 95.7 % of boys are moderately physically active at least 60 min a day at the age of 6 years, but at the age of 15 years only 43.2 % of girls and 58.1 % of boys reach this level of PA [6]. Regular PA may improve self-esteem [7] and reduce symptoms of anxiety and depression [8]. Furthermore, participation in PA is positively associated to academic performance in children and adolescents [9, 10], and physically active adolescents tend to be physically active into adulthood [11]. A recently published review [12] showed that girls are less physically active than boys, and that some physical education (PE) programs not effectively meet the girls’ needs. However, a qualitative study indicates that many of the barriers to activity are not gender specific, but are related to perceived lack of competence [13].

Schools are essential in achieving health literacy in a population, and contribute to the achievement of public health goals in conjunction with their educational commitments [14]. Physical education in schools is universally applicable, and important for fundamental movements’ skills and as a basis for adult health behaviours [15]. A review by Dobbins et al. [16] concluded that school-based programs might increase the students’ moderate-to-vigorous intensity physical activity (MVPA) level. Focus on curriculum, policy and environmental strategies as key aspects of an ecological approach to increase MVPA appear to be more effective than a curriculum-only approach [17]. It has been recommended that PA programs should involve PE teachers, support from family and community stakeholders, as well as considerations about how the physical environment facilitates PA [18, 19]. For adolescents, multicomponent interventions including both school and family or community involvement facilitate improvement in levels of PA [20].

Population levels of PA depend on economic conditions and societal norms [21]. A report from the World Health Organization describes that adults with vocational education are less physically active than adults with college or university background in developed countries [22]. Thus interventions intending to increase PA among children and adolescents should especially focus on students attending vocational studies.

In review studies it has been suggested that PA programs should encourage adolescents to take charge of program development, selecting activities that generate fun, enjoyment and interest [23, 24]. Self-determination Theory (SDT) suggests autonomy, perceived competence and relatedness as fundamental psychological needs [25]. Students enjoy participation in PE when they have a choice of activities, feel competent, in control and supported by their peers and teachers [13]. PE programs based on these principles will support intrinsic motivation and adherence to PA [26, 27].

School-based interventions that enhance active participation in PE have shown positive outcomes on PA maintenance in children and adolescents [15]. The intention of PE programs in Norwegian high schools is to inspire the students to experience enjoyment and lifelong adherence to participation in PA, and develop a positive perception of body, self and identity [28]. To reach this goal, design of PE programs should intend to facilitate adherence to PA in general. In Norway, vocational students attend PE less frequently; have lower grades in PE and more frequently drop out of school than other high-school students [29]. Public health nurses and PE teachers frequently meet these students to discuss health issues and participation in PE class. In the present study we intended to explore PE teachers’ and public health nurses’ perceptions of high school students’ attitudes towards participation in PE in general, and to explore PE teachers’ thoughts about how to facilitate and promote students’ participation in PE.

## Methods

### Design

Prior to an initiative from PE teachers, introducing a new PE model in August 2013, PE teachers and public health nurses from two high schools in Kristiansand municipality, Vest-Agder County in southern Norway, participated in focus-group interviews. A focus group study was chosen to gain insight from several participants, and from discussions among them. The participants shared information about their perception of students’ participation in PE programs and health behaviour. In the new model the students chose participating in one out of two alternative educational programs; 1) “sports enjoyment” focusing on PA skills, technique and improvement of physical performance, and 2) “motion enjoyment” focusing less on technique and physical performance, but more on health and facilitating positive experience when participating in PE lessons.

A total of 830 boys and girls started in the two high schools in 2013. A total of 181 first year students comprising 141 (77.9 %) girls and 40 (22.1 %) boys attending to vocational studies of Restaurant and Food Processing (*n* = 58), Design, Arts and Crafts (*n* = 60) or Healthcare, Childhood and Youth Development (*n* = 102) were recruited for participation in the new PE program. The majority (69.8 %) of the students were adolescents aged 15 – 16 years old, and the mean age was 17 (SD = 2.6; range 15 – 31 years).

### Participants

Before implementation of the new model, 1 focus group interview was conducted with a strategic sample of 6 PE teachers (1 male, 5 female). One focus group interview was conducted with 8 public health nurses (all female) representing 4 different high schools. At the end of the first year after implementation of the new model, we conducted 2 additional focus group interviews with 10 PE teachers (3 male, 7 female) representing the 2 high schools that had tried out the new model.

### Analysis

We audiotaped and transcribed the audiotapes verbatim. In analyses we used Systematic Text Condensation and an editing analysis style [30, 31]. Bracketing preconceptions, two researchers (EA and GR) independently read the material searching for an overall impression and established preliminary subgroups. We then examined the text for units of meaning representing information about perception of students’ participation in physical education. In an iterative process we coded and grouped these units, contrasted and abstracted the content in each group, and finally discussed and summarized the content of each group into generalized descriptions. To support analysis we created mind maps, and discussed the analysis at each step to reach agreement. Quotations were used to illustrate and support findings. Intending member checking, we presented the results to a group of PE teachers and public health nurses. More information about methods is available from the interview guide and a COREQ checklist [32] (Appendices 1 and 2).

### Ethics

Participation in the focus groups was based on a written consent. The study was approved by the Norwegian Social Science Data Services (35639) and by the Norwegian Regional Ethics Committee, South-East B (2013/1235).

## Results

To a large extent, public health nurses and PE teachers agreed upon high school students’ attitudes towards participation in physical education. The PE teachers agreed about how to promote students participation in PE, but expressed somewhat different experiences with the new PE model, depending on their previous education models. We did not find major differences in statements from male and female teachers. All results are based on information provided from the participants taking part in the focus groups. The headings and subheadings correspond to themes and subthemes identified in the analysis.

### Part of a more complex picture

The participants stated that students, in general, were engaged and appreciated participating in PE lessons. A higher number of students attending vocational programs were reported to skip PE classes compared to students attending general programs. Many of them prepared to enter occupations that encompassed great demand on physical fitness. More girls than boys did not participate in PE classes. Those who seldom attended often were reported to have problems with other subjects as well, and more often there was no basis for assessment. Some of these students were characterized as outsiders in several arenas, and some of them had been bullied or had other adverse childhood experiences. Anxiety, depression, eating disorders and other mental health complaints were reported as common among girls in this group. Use of illegal drugs was reported to be more common among seldom attenders to PE class. The participants reported that students who seldom participated in PE classes more frequently than other students expressed low self-efficacy.

What characterize these students are anxiety and other mental problems, this may be the basic problem for many of them. Some lack motivation, not only regarding PE, but other subjects as well. (Female teacher)

The participants stated that some students suffered from serious physical diseases. More often, however, especially female students who skipped PE class reported medically unexplained physical symptoms. PE teachers as well as public health nurses related these students’ health behavior to a social heritage characterized by a lack of initiative and a weak attachment to work life among their mothers. Lack of personal and family structure and role models was believed to have a negative impact on lifestyle. This included diet, sleep habits, time spent on gaming and follow up of school work as well as attending to PA. Split families were believed to imply practical challenges, such as remembering to bring training gear to PE class. Several seldom attenders were reported to smoke more frequently, and also needed individually tailored education in other subjects.

They have never submitted homework or written reports in school. Nothing…. They are completely without initiative. (Female public health nurse)

### Health, body and body image dissatisfaction

The participants expressed ambiguity among female students concerning body exposition. On one-hand students dressed sexually provocatively. On the other hand, the same students were reluctant to expose their bodies to fellow female students when showering naked after PE lessons. Students suffering from anorexia and scars due to self-injury were believed to be ashamed to expose their body in public. Public health nurses told that students who seldom attended PE class frequently visited the school health services.

They wear skirts just below their buttocks. They have large necklines. They just lean forward, and you can observe most of it. But when it comes to showering naked, it is something else in a way. (Female public health nurse)

The PE teachers reported that obese boys with impaired motoric skills less often attended to PE lessons. Among non-western immigrants, boys more often than girls were reported to take part in physical education. Limitations due to girls ‘outfit and acceptability to expose body surface among some religious groups was suggested as an explanation.

The participants stated that many students had unhealthy dietary habits, including an irregular meal pattern, which may have negatively affected their energy level and participation in PE classes. According to the participants, boys studying building and construction often had sugar-sweetened soft drinks (Coca-Cola) and buns for breakfast. Students, who lacked a social meal framework at home, were reported to have insufficient energy level and complained about headache and nausea.

Quite a few of them lack meal structure completely. Many have a single parent, and have never shared a meal. It is just a quick meal on the worktop in the kitchen. (Female public health nurse)

The public health nurses reported that some students did not seem to understand the connection between a healthy diet, body function and physical fitness.

### Previous experiences with physical activity and education

Although the majority of students were clever and engaged in PE class, the PE teachers reported that many students lacked basic knowledge about physical fitness and motoric skills to be able to master daily activities in PE class. The PE teachers believed this was a result of disengaged physical education teachers and low expectations concerning attendance, learning outcome and physical skills in junior high school. Some students had not attended to PE for several years with no obvious explanation, and the PE teachers reported that parents had approved this behavior and not expected their children to participate.

They have always slipped away, and it has not been a problem. Mom or dad has signed a note, and even in junior high school they have made arrangements so that they did not attend. (Female public health nurse)

The PE teachers experienced that students, in general, paid less attention to PE than to other subjects, and expected a limited learning outcome.

The participants stated that several students quit participation in organized sport activities before starting high school. They suggested a focus on sports performance rather than participation and enjoyment at an early age, and lack of parental engagement as an explanation. Some of the students were reported to exercise at health clubs, but few practiced active commuting.

They just wait for the bus outside school … wait and wait and wait. And they simply plan to head for the end of the street to catch another bus. They could have been there much earlier if they had walked, but that seems unthinkable to many of them. (Female PE teacher)

Outdoor physical environmental factors that stimulated physical activity were reported common in junior high school, but usually absent in high school. The students were experienced as group members, and often adopted the same behavior as their friends concerning participation in PE.

### The PE Teacher’s role

The public health nurses underscored the importance of the high school PE teachers’ engagement and willingness to invent tailored solutions for individual students as well as groups. They told about students that experienced their teachers in high school as role models and source of inspiration for participation in PE class.

He should be cloned. He is able to inspire girls that never have participated to experience PE as the funniest subject at school. (Female public health nurse)

On the other hand, the public health nurses, as well as the PE teachers, described that several of the students had experienced unengaged teachers who did not actively participate in class. This was not believed to facilitate education to support the students’ needs and motivation to participate in PE class.

The PE teachers wished to provide knowledge and let the students experience mastering physical activity. To succeed, professional knowledge and engagement was considered as important. The PE teachers also wished to pay attention to students who enjoyed taking part in PE, and at the same time being aware of individual needs of those with less engagement. This was challenging with many students in each PE class.

I try a calm and friendly approach, ask them what they can do, how much they can take part, let them be responsible. I don’t focus on grades and pressure and all that. And now she is participating in class now and then. (Female PE teacher)

The PE teachers expressed significant solidarity to their peers, and acted as a group to promote physical education as an important subject in the school’s curriculum.

### Facilitation and choice

The PE teachers reflected on how to approach and follow-up seldom attenders in PE classes. They expressed ambiguity regarding the framework decided by the PE curriculum with respect to being able to provide a variety of activities, and to give grades. They discussed whether PE should provide knowledge about health or focus on movement skills. Basic physical fitness and variation was considered necessary to initiate PE that provided the experience of mastering and enjoyment.

All participants stated that it was a success to let the students choose among different options of activities and skills level. This implied that more students participated, coped and seemed to have fun. Students who did not like taking part in ordinary PE appreciated alternatives like yoga, swimming or climbing.

I have taught those who never have participated in physical education, those who hated it. At the end of the term they experiences self-efficacy and enjoyed taking part in class. Not scary anymore. It is a bit fun when they give such feedback. (Female PE teacher)

When the students could choose their PE program, the PE teachers experienced more homogenous groups with respect to movement skills, and better flow in performance in class. The participants reported that the students did not devaluate each other based on their choice of PE program. A separate group for students with injuries or other health issues facilitated individual tailored activities.

The groups are more homogenous, and when you plan and carry out education the students are more satisfied. (Male PE teacher)

One of the schools arranged informal table-tennis tournaments, dance battles and zumba-dance as an open offer to all students in addition to PE lessons. The students seemed to enjoy and engage in these activities, even students who did not take part in ordinary PE lessons.

## Discussion

High school students were reported to be a heterogeneous group with respect to PE skills, preferences, previous experiences and possibly interfering health issues. Thus, possible explanations of high school students’ attendance and motivation for PE are probably complex, and skipping PE class may represent one piece of a larger picture. Factors that influence the PA among adolescents are numerous. In a review study, variables that were consistently associated with adolescents’ PA were gender (male), ethnicity (white), age (inverse), perceived activity competence, intentions, depression (inverse), previous physical activity, community sports, sensation seeking, sedentary after school and on weekends (inverse), parent support, support from others, sibling physical activity, direct help from parents, and opportunities to exercise [33].

In the present study, PE teachers as well as public health nurses reported that students who seldom attend PE class often strived with other aspects of life and other subjects at school, and that participation in PA was related to self-rated health and body image satisfaction. In a study of Norwegian adolescents, Dyremyhr et al. [34] reported that PA was positively related to self-reported health, but had negative associations with body image. These results are in line with findings from the present study, where the participants also reflected upon how to understand and handle body dissatisfaction and the students’ ambiguity of exposing their body in PE class. Low body satisfaction has been identified to predict behaviors that may increase risk for weight gain and poorer overall health [35]. Furthermore, in a recently published longitudinal study high levels of body dissatisfaction throughout adolescence (14–18 years of age), especially among girls was reported [36]. The latter mentioned study also reported that a concern about weight at age 14 was associated with increased body dissatisfaction four years later.

The PE teachers in our study experienced a lack of competence concerning fundamental movement skills among students, and low expectations of learning. Many students had been allowed to skip PE in junior high school without obvious reason. Basic movement skills are essential to master PA, and positively associated with PA and fitness levels [37]. The PE teachers emphasized the importance of competence and engagement of PE teachers in junior high school. The public health nurses underscored the importance of PE teachers as role models for the students. Perceived lack of competence may leave students embarrassed, humiliated and reluctant to engage in PE [13]. Competent PE teachers significantly improve fundamental movement skill proficiency in youth [37], and perceived competence is essential to facilitate self-determination and autonomous motivation for participation in physical education [38]. In Norwegian junior high schools 72.0 % of PE teachers have formal PE competence, but only 44.0 % have studied PE one year or more [39].

The PE teachers taking part in the present study experienced that giving their students an influence on the content of PE facilitated engagement and participation in PE class. They also appreciated having a possibility to tailor their education to fit the needs, preferences and skills of their students without compromising with the framework of the education program. Individual preferences and experiences as well as social environmental factors may influence intrinsic motivation for participation in physical education [40]. PE programs that offer choice of activities and supports individual needs are probably beneficial to support adoption of PA [23, 24]. A model of PE that supports perceived autonomy, develop and expand the students’ competence and positively influence the students’ relation to peers and teachers comply with the basic psychological needs described in SDT [25]. A PE model that supports self-determination is likely to facilitate a sustainable healthy lifestyle, and is positively related to self-rated health [26, 27].

In the present study the participants expressed a nuanced description of why some students didn’t attend to PE. However, they also provided quite rough and stereotypical descriptions of why some students did not participate in PE classes as expected. Communication in focus groups is often informal when the participants know each other [41]. Dialogues among peers in a safe environment may be described as back-stage communication, and probably exist in every trade when peers meet, share experiences, and have discussions not meant for outsiders [42, 43]. Information based on dialogues among peers may support information from observational studies and questionnaires, and nuance the picture of professionals’ perceptions and attitudes.

### Strengths and weaknesses

The present study is based on information from public health nurses and physical education teachers. Thus, information about background, attitudes and behaviors of the target group is limited. The students’ perspectives were not included. The study design may imply that the participants adjusted their utterances to the presence of the researchers. However, the presence of rough characteristics of students may indicate that this influence was limited. Several measures have been used to strengthen the consistency of the findings, including member checking, methods triangulation and observer triangulation. The study adds information about how public health nurses and PE teachers in high schools perceive students’ attitudes towards PE programs, and indicate willingness to include students’ perspectives in designing PE programs.

#### Implications for practice

The results of the present study are relevant to Norwegian high schools, and to high schools in societies with a similar educational system. It would be of interest to study the effect of a PE model as described on long term PA levels in a longitudinal study. The study may provide information about how PE in Norwegian high schools is perceived by PE teachers and public health nurses, and suggests a framework of PE that may facilitate that more students enjoy and take part in regular PA.

## Conclusion

PE teachers and public health nurses suggested that high school students’ attitudes towards participation in PE is heterogeneous, and depends on the students’ previous experiences with PE and PE teachers, and on their present health and quality of life. PE teachers as well as public health nurses accept and recommend PE programs that encourage the adolescents to take part in program development, selecting activities that generate competence, fun and enjoyment.

## Appendix 1: Interview guide

### What is your impression of how students experience to be physically active?

- Health and physical activity literacy
- Fitness skills
- Experiences from PE in junior high school
- How do they experience to expose their body?

### Who are the students who seldom attend to PE class?

- What characterizes the students?
- What do they study?
- What seems to be their problems?
- Gender?

### How do you experience meeting these students?

- How do you choose to meet them?

### How have you facilitated the PE activities to include students who seldom take part in PE class?

- What is desirable?
- What is possible to do?

### What is important to include in a PE model intending to include more students?

- Mastering
- Enjoyment
- What du you think about the new PE model used in this project?

### Appendix 2

**Table 1:** Consolidated criteria for reporting qualitative studies (COREQ): 32-item checklist

No. item	Guide questions/Description	Response
Domain 1: Research team and reflexivity		
Personal Characteristics		
1. Inter viewer/facilitator	Which author/s conducted the interview or focus group?	EA, THS, CSO and GR conducted the focus groups.
2. Credentials	What were the researcher’s credentials? E.g. PhD, MD	EA: MD, PhD. THS: MSc, PhD. SB: MSc, PhD. CSO: MSc GR: MSc, PhD
3. Occupation	What was their occupation at the time of the study?	EA: Public health officer, THS: Associate professor, SB: Professor, CSO: Physiotherapist, GR: Associate professor
4. Gender	Was the researcher male or female?	Female: THS, CSO, GR. Male: EA, SB
5. Experience and training	What experience or training did the researcher have?	The group of researchers had experience with qualitative and quantitative research methods based on several previous research projects.
Relationship with participants		
6. Relationship established	Was a relationship established prior to study commencement?	The PE teachers initiated the contact with the researchers based on their interest of trying out a new PE model.
7. Participant knowledge of the interviewer	What did the participants know about the researcher? e.g. personal goals, reasons for doing the research	The participants knew that members of the research group were interested in adolescent health.
8. Interviewer characteristics	What characteristics were reported about the inter viewer/facilitator? e.g. Bias, assumptions, reasons and interests in the research topic	The interviewers represented different professions. Medicine (EA), nutrition science (THS), sports science (SB), physiotherapy (CSO) and nursing science (GR).
Domain 2: study design		
Theoretical framework		
9. Methodological orientation and Theory	What methodological orientation was stated to underpin the study? e.g. grounded theory, discourse analysis, ethnography, phenomenology, content analysis	Systematic Text Condensation represents a hermeneutic phenomenological approach. Self-determination theory was used as a theoretical framework of the study.
Participant selection		
10. Sampling	How were participants selected? e.g. purposive, convenience, consecutive, snowball	We invited all PE teachers in high schools in Kristiansand municipality and public health nurses in high schools in Kristiansand and Mandal municipality to take part in the study by purposeful sampling.
11. Method of approach	How were participants approached? e.g. face-to-face, telephone, mail, email	The participants were approached by email.
12. Sample size	How many participants were in the study?	Information is given in the methods chapter.
13. Non-participation	How many people refused to participate or dropped out? Reasons?	Public health nurses and PE teachers from one high school were not able to participate due to other obligations. No one refused to participate.
Setting		
14. Setting of data collection	Where was the data collected? e.g. home, clinic, workplace	The interviews took place at regular meetings at the schools.
15. Presence of non-participants	Was anyone else present besides the participants and researchers?	No.
16. Description of sample	What are the important characteristics of the sample? e.g. demographic data, date	Both male and female PE teachers participated. Only female public health nurses worked at the high schools.
Data collection		
17. Interview guide	Were questions, prompts, guides provided by the authors? Was it pilot tested?	The interview guide is enclosed with the manuscript.
18. Repeat interviews	Were repeat inter views carried out? If yes, how many?	Information is provided in the methods chapter.
19. 19. Audio/visual recording	Did the research use audio or visual recording to collect the data?	The interviews were audiotaped.
20. Field notes	Were field notes made during and/or after the inter view or focus group?	Short field notes were made after the interviews.
21. Duration	What was the duration of the inter views or focus group?	The duration of the interviews were 60–90 min.
22. Data saturation	Was data saturation discussed?	Data saturation was discussed and considered sufficient to perform the analysis.
23. Transcripts returned	Were transcripts returned to participants for comment and/or correction?	The transcripts were not returned to the participants, but the preliminary findings were presented to and discussed with the participants.
Domain 3: analysis and findings		
Data analysis		
24. Number of data coders	How many data coders coded the data?	Two researchers (EA and GR) coded the data.
25. Description of the coding tree	Did authors provide a description of the coding tree?	The headlines and subtitles in the results presentation represent the final coding tree.
26. Derivation of themes	Were themes identified in advance or derived from the data?	Themes were derived from the data.
27. Software	What software, if applicable, was used to manage the data?	We used NVivo® for Mac version 10.2.1 to assist analysis.
28. Participant checking	Did participants provide feedback on the findings?	Yes, as reported in the methods chapter.
Reporting		
29. Quotations presented	Were participant quotations presented to illustrate the themes/findings? Was each quotation identified? e.g. participant number	Yes. Gender and profession identified the participants.
30. Data and findings consistent	Was there consistency between the data presented and the findings?	Yes.
31. Clarity of major themes	Were major themes clearly presented in the findings?	Yes.
32. Clarity of minor themes	Is there a description of diverse cases or discussion of minor themes?	Several diverse cases and minor themes are described in the results chapter.

Developed from:

Tong A, Sainsbury P, Craig J. Consolidated criteria for reporting qualitative research (COREQ): a 32-item checklist for interviews and focus groups. International Journal for Quality in Health Care. 2007. Volume 19, Number 6: pp. 349 – 357
